# Protective Effects of Simvastatin, a Lipid Lowering Agent, against Oxidative Damage in Experimental Diabetic Rats

**DOI:** 10.1155/2011/167958

**Published:** 2011-12-06

**Authors:** Ahmed M. Mohamadin, Ahmed A. Elberry, Hala S. Abdel Gawad, Gehan M. Morsy, Fahad A. Al-Abbasi

**Affiliations:** ^1^Department of Chemistry for Health Sciences, Deanery of Academic Services, Health Sciences Track, Taibah University, Al-Madinah, Saudi Arabia; ^2^Biochemistry Department, Faculty of Pharmacy, Al-Azhar University, Nasr City, Cairo, Egypt; ^3^Department of Clinical Pharmacy, Faculty of Pharmacy, King Abdulaziz University, Jeddah, Saudi Arabia; ^4^Department of Physiology, College of Medicine, Taibah University, Al-Madinah, Saudi Arabia; ^5^Biochemistry Department, Applied Science College, Taibah University, Al-Madinah, Saudi Arabia; ^6^Department of Biochemistry, Faculty of Science, King Abdulaziz University, Jeddah, Saudi Arabia

## Abstract

The present study was undertaken to evaluate the possible protective effects of simvastatin (SMV) against oxidative stress in streptozotocin- (STZ)-induced diabetic rats. Diabetes was induced experimentally in rats by i.p. injection of STZ in a dose of 60 mg/kg bwt. After 5 weeks of STZ injection, there were apparent reductions in the animal body weight and significant increase in blood glucose, HbA1_c_, urea, creatinine, AST, ALT, and lipid profiles with a concomitant decrease in total hemoglobin, plasma glutathione and vitamin C as compared to the control group. The treatment with SMV at a dose (10 mg/kg, orally) normalized all the above-mentioned biochemical parameters in STZ-induced diabetic rats. *In vitro* studies confirmed the free radical scavenging and antioxidant activity of SMV. Therefore, the present results revealed that SMV has a protective effect against STZ-induced oxidative damage by scavenging the free radicals generation and restoring the enzymatic and nonenzymatic antioxidant systems.

## 1. Introduction

Diabetes is a major threat to global public health, and the number of diabetic patients is rapidly increasing world-wide. More than 220 million people worldwide have diabetes and this number is likely to be more than double by the year of 2030 [1]. Apart from this, more than 60% of the world population with diabetes will come from Asia [2]. It has already been established that chronic hyperglycemia of diabetes is associated with long-term damage, dysfunction, and eventually failure of organs, especially the kidneys, nerves, heart, eyes, and blood vessels [3]. About 50% of individuals with diabetes are affected with one or more of the above complications. 

Oxidative stress plays an important role in chronic complications of diabetes and is postulated to be associated with increased lipid peroxidation (LPO) [4, 5]. Streptozotocin (STZ) is frequently used to induce diabetes mellitus in experimental animals through its toxic effects on pancreatic *β*-cells [6]. The cytotoxic action of STZ is associated with the generation of reactive oxygen species (ROS) causing oxidative damage [7]. Oxidative stress increases due to several factors: enhancement of glucose auto-oxidation, stimulation of the polyol pathway, production of advanced glycation products, and reduction in antioxidant defenses, such as depletion of cellular antioxidant levels and decreased antioxidative enzyme activity [3, 8]. In diabetes there are significant changes such as increased LPO, dyslipidemia, and irregularities in the metabolism of proteins, lipids, and carbohydrates [9]. Chemicals with antioxidant properties and free radical scavengers may help in the regeneration of *β*-cells and protect pancreatic islets against cytotoxic effects of STZ [6, 10].

3-Hydroxy-3-methylglutaryl coenzyme A (HMG-CoA) reductase inhibitors (statins) including simvastatin (SMV) have been previously demonstrated in diabetic patients with their ability to reduce albuminuria [11–13]. Furthermore, administration of statins in experimental diabetes has previously been reported to be associated with a reduction in the renal expression of the prosclerotic cytokine, transforming growth factor-*β*1 (TGF-*β*1) [14, 15], improve wound healing [16], and improve endothelial function in experimental diabetic rats [17]. Furthermore, a meta-analysis provided evidence that statins reduce the progression of retinopathy [18] and nephropathy [19].

One of the pleiotropic mechanisms receiving much attention is the antioxidant effect of statins [20, 21]. Mechanisms for this may be due to the inhibition of oxidant formation by affecting NADPH-oxidase, blocking of the effects of ROS by upregulation of antioxidant enzymes, or an increase in nitric oxide bioavailability which neutralizes radicals [22]. ROSs, including free radicals such as HO^•^ and O_2_^∙−^, and molecules such as hydrogen peroxide, are involved and contribute to the development of atherosclerosis [23]. Important sources of ROS are NADPH oxidases from endothelial cells, smooth muscle cells, fibroblasts, and infiltrated monocytes/macrophages [24]. Therefore, the present study was designed to investigate the effects of simvastatin on oxidative stress markers as well as on the antioxidative defense system in STZ-induced diabetic rats.

## 2. Materials and Methods

### 2.1. Chemicals

Simvastatin (SMV), Streptozotocin (STZ), hydrogen peroxide, glutathione, 5,5′-dithio-*bis*-(2-nitrobenzoic acid), thiobarbituric acid (TBA), 1,1,3,3-tetraethoxypropane, glutathione-reduced form, glutathione reductase, oxidized glutathione (GSSG), NADPH-tetra salt, and ethylenediamine tetra acetic acid (EDTA) disodium salt were purchased from Sigma-Aldrich Chemical (St Louis, MO, USA). Catalase (CAT), superoxide dismutase (SOD), and glutathione peroxidase (GSH-Px) reagent assay kits were purchased from Cayman chemical (MI, USA). Glucotest (glucose urine strips) was purchased from Roche Diagnostics (Mannheim, Germany). All other chemicals were of the highest grade available commercially.

### 2.2. Experimental Animals

Forty male Sprague-Dawley rats, weighing 200–220 g, were obtained from King Fahd Medical Research Center, King Abdulaziz University (Jeddah, Saudi Arabia). Guides for the care and use of laboratory animals were approved by the local ethics committee at the King Abdulaziz University. Rats were housed in wire-floored cages under a 12 h light-dark cycle for at least 7 days prior to treatment and were fed standard laboratory chow and tap water *ad libitum*. The room temperature was kept at 22 ± 2°C. All stressful conditions were avoided. Rats were fasted overnight prior to the study and housed in mesh-bottomed cages to minimize coprophagia. Except for the last hour, water was supplied *ad libitum*.

### 2.3. Experimental Induction of Diabetes

Animals were fasted overnight and diabetes was induced by a single intraperitoneal injection of a freshly prepared solution of STZ (60 mg/kg) in 0.1 mol/L citrate buffer (pH 4.5) [25]. The dosing volume was 1 mL/kg. To prevent fatal hypoglycemia, rats were kept on 5% glucose solution for 24 h after STZ injection. Successful induction of diabetes was confirmed by measuring the fasting blood glucose concentration in rats 6 h after injection of STZ. Rats with a fasting blood glucose level >250 mg/dL were considered diabetic and included in the present study.

### 2.4. Experimental Design

One week after the administration of STZ and citrate buffer, control and diabetic rats were randomly assigned to treatments with SMV or glibenclamide. Rats were divided into five groups, with eight rats in each group, as follows: (i) Group I, control rats receiving vehicle solution (citrate buffer; 1 mL/kg/day); (ii) Group II, control rats receiving SMV (10 mg/kg/day) only; (iii) Group III, diabetic control rats injected with STZ (60 mg/kg bodyweight, i.p.) only; (iv) Group IV, diabetic rats treated with SMV (10 mg/kg/day) in aqueous solution via an intragastric tube 3 days after STZ treatment and continued for 5 weeks [26]; and (v) Group V, diabetic rats treated with glibenclamide (0.60 mg/kg/day) in aqueous solution daily via an intragastric tube for 5 weeks.

The following parameters were assessed in each of the study groups during the treatment period: daily fluid and food consumption, weekly body weight, and blood glucose concentration (Glucostix strips tested in a glucometer; Abbott Laboratories, Medisense Products, Bedford, MA, USA). Food consumption was determined by subtracting leftovers from the diet provided to rats at 2-day intervals.

After the last treatment (5 weeks), rats were fasted overnight and sacrificed by cervical decapitation. Blood was collected in two separate tubes, with and without anticoagulant, for the estimation of glucose, hemoglobin, HbA1_c_, lipid profiles, liver and kidney function tests. Liver, and kidney tissues were excised immediately from the rats and stored in ice-cold containers.

### 2.5. Preparation of Liver and Kidney Homogenates

After blood sample collection, the animals were killed and then the liver and kidneys were removed immediately and placed in ice-cold 0.1 M phosphate buffer saline (PBS) with pH 7.5. The tissues were then blotted dry and weighted. A 10% tissue homogenates (w/v) of the liver and kidney were prepared in PBS. Homogenates were then centrifuged at 1000 rpm for 10 min in a cooling centrifuge to remove the cell debris. Then the supernatants were placed in −80°C until further use to determine antioxidant enzymes activities and LPO. Protein was estimated by the method of Lowry et al. [27].

### 2.6. In Vitro Antioxidant Study

#### 2.6.1. Total Antioxidant Activity Determination

The antioxidant activity of SMV was determined according to the thiocyanate method [28]. Ten milligrams of SMV was dissolved in 10 mL water. SMV at various concentrations (25, 50 and 75 *μ*g/mL) or standard sample (*α*-tocopherol) in 2.5 mL of potassium phosphate buffer (0.04 M, pH 7.0) was added to linoleic acid emulsion. Five-milliliter linoleic acid emulsion consists of 17.5 *μ*g tween-20, 15.5 *μ*L linoleic acid, and 0.04 M potassium phosphate buffer (pH 7.0). On the other hand, 5.0 mL control consists of 2.5 mL linoleic acid emulsion and 2.5 mL potassium phosphate buffer (0.04 M, pH 7.0). The mixed solution was incubated at 37°C in a glass flask and in the dark. After the mixture was stirred for 3 min, the peroxide value was determined by reading the absorbance at 500 nm in a spectrophotometer, after reaction with FeCl_2_ and thiocyanate at intervals during incubation. During the linoleic acid oxidation, peroxides formed. These compounds oxidize Fe^2+^ to Fe^3+^. The latter Fe^3+^ ions form complex with SCN^−^, which had maximum absorbance at 500 nm. Therefore, high absorbance indicates high linoleic acid oxidation. The solutions without SMV or standards were used as blank samples. All data about total antioxidant activity are the average of duplicate analyses. The inhibition of LPO in percentage was calculated by following equation:

(1) $$\text{\%}\;\text{Inhibition}=\left[\frac{\left(A_{0}-A_{1}\right)}{A_{0}}\right]\times 100,$$

where *A*_0_ was the absorbance of the control reaction and *A*_1_ was the absorbance in the presence of the sample of SMV. 

#### 2.6.2. DPPH Scavenging Activity

The free radical scavenging activity of SMV, buthylated hydroxyanisole (BHA), and *α*-tocopherol were measured using the method of Shimada et al. [29] with a slight modification. A 0.1 mM solution of 1,1-diphenyl-2-picryl-hydrazyl (DPPH^•^) in ethanol was prepared and 1 mL of this was added to 3 mL of SMV solution in ethanol at different concentrations (20–80 *μ*g/mL). After 30 min, a decrease in absorbance was measured at 517 nm and the actual decrease in absorption induced by the test compound was calculated by subtracting that of the control.

### 2.7. Biochemical Analysis

#### 2.7.1. Plasma Glucose Assay

Plasma glucose levels were estimated using a commercial kit (Sigma Diagnostics Pvt. Ltd., Baroda, India) by the method of Trinder [30].

#### 2.7.2. Total Hemoglobin (Hb) and Glycosylated Hemoglobin (HbA1_c_) Assays

Total Hb and HbA1_c_ were estimated by Diagnostic kit-Bio Systems (Costa Brava, Spain).

#### 2.7.3. Plasma Lipid Profile

Plasma total cholesterol (TC), triglycerides (TGs), and HDL-cholesterol (HDL-C) levels were determined by enzymatic colorimetric methods. The TC concentration was proportional to the dye product formed by the reaction of the hydrogen peroxide released with 4-aminophenazone and phenol reagent, measured at 540 nm. Plasma HDL-C was determined by precipitation with phosphotungstate-MgCl_2_ solution [31]. The enzymatic method used for determination of cholesterol in the supernatant used kits (Cholesterol Oxidase/Peroxidase kit) is supplied by BioSystems (Barcelona, Spain). Determination of TG level was based on TG hydrolysis by lipase, where the glycerol formed used glycerol kinase, phosphoglycerol oxidase, and peroxidase to form hydrogen peroxide, which reacts with 4-aminophenazone and 4-chlorophenol to produce a complex that can be measured at 620 nm using an autoanalyser (model 7150; Hitachi, Tokyo, Japan).

#### 2.7.4. Liver Function Tests

The activities of serum hepatic marker enzymes, namely, aspartate aminotransferase (AST) and alanine aminotransferase (ALT) were assayed in serum using standard kits from Merck using colorimetric method [32]. The results were expressed as U/L.

#### 2.7.5. Kidney Function Tests

Serum creatinine and urea were determined at 37°C colorimetrically by the modified Jaffe method and the modified Berthelot-Searcy enzymatic method, respectively. They were assayed using reagents obtained from assay kits (Quimica Clinica Applicada, Spain). Urinary protein was quantified by the Biuret method using bovine serum albumin as the standard.

#### 2.7.6. Determination of Plasma Antioxidants

Reduced glutathione (GSH) was estimated by the method of Ellman [33]. A 0.1 mL of plasma was precipitated with 5% TCA. The contents were mixed well for complete precipitation of proteins and centrifuged. To an aliquot of clear supernatant, 2.0 mL of 0.6 mM 5,50-dithiobis-2-nitrobenzoic acid (DTNB) reagent and 0.2 M phosphate buffer (pH 8.6) were added to obtain a final volume of 4.0 mL. The absorbance was read at 412 nm against a blank containing 5% TCA instead of sample. A series of standards treated in a similar way were also run to determine the glutathione content. The amount of glutathione was expressed as mg/dL in plasma.

Ascorbic acid (Vitamin C) concentration was measured by the method of Omaye et al. [34]. To 0.25 mL of plasma, 0.75 mL of 6% TCA was added and centrifuged (3500 g, 20 min). To 0.25 mL of supernatant, 0.25 mL of dinitrophenylhydrazone (DNPH) reagent (2% DNPH and 4% thiourea in 4.5 M sulphuric acid) was added and incubated for 3 h at room temperature. After incubation, 1.25 mL of 85% sulphuric acid was added and color developed was read at 530 nm after 30 min.

#### 2.7.7. Lipid Peroxidation (LPO) Assay

LPO was determined by measuring thiobarbituric acid reactive substances (TBARS) content in tissue homogenates according to the method of Uchiyama and Mihara [35], with some modifications. Briefly, 0.01 g liver or kidney tissue was homogenized with 0.9 mL of 1.15% KCl solution and the TBARS content was measured spectrophotometrically at 532 nm. The TBARS content was calculated based on a standard curve using 1,1,3,3-tetraethoxypropane as a standard.

#### 2.7.8. Reduced Glutathione (GSH) Assay

GSH was measured in liver and kidney tissue homogenates by the reaction of the sulphydryl groups (SH) in the nonprotein fractions with 5,5-dithiobis-(2-nitrobenzoic acid; DTNB or Ellman's reagent). The GSH levels were compared with a standard curve prepared using different known concentrations of GSH. The product was measured spectrophotometrically at 412 nm [33].

#### 2.7.9. Enzymatic Antioxidants Assays

Superoxide dismutase (SOD) activity was determined in homogenates and erythrocytes according to the methods of S. Marklund and G. Marklund [36]. A colorimetric assay was performed that involved generation of superoxide by pyrogallol auto-oxidation and the inhibition of superoxide-dependent reduction of the tetrazolium dye 3-(4,5-dimethylthiazol-2-yl) 2,5-diphenyltetrazolium bromide (MTT) to formazan by SOD, measured at 570 nm. The amount of MTT formazan was calculated by using a molar extinction coefficient *E*570 of 17 000 L/mol per cm. One unit of SOD was defined as the amount of protein required to inhibit MTT reduction by 50%.

Catalase (CAT) activity was measured according to the method described by Aebi [37]. One unit of CAT activity was defined as the amount of enzyme required to decompose 1 mmol H_2_O_2_ in 1 min. A 50 mL aliquot of tissue supernatant was added to a cuvette containing 1.95 mL of 50 mmol/L phosphate buffer (pH 7.0). The reaction was started by the addition of 1.0 mL freshly prepared 30 mmol/L H_2_O_2_. The rate of decomposition of H_2_O_2_ was measured spectrophotometrically at 240 nm for 1 min.

The activity of glutathione peroxidase (GSH-Px) was determined according to the method of Lawrence and Burk [38]. The assay mixture consisted of 2.0 mL of 75 mmol/L phosphate buffer (pH 7.0), 50 mL glutathione, 0.1 mL of 30 units/mL glutathione reductase, 0.1 mL of 15 mmol/L EDTA, 0.1 mL of 3 mmol/L NADPH, and the appropriate amount of tissue supernatant to reach a final volume of 3.0 mL. The reaction was started by the addition of 0.1 mL of 7.5 mmol/L H_2_O_2_. The rate of change of absorbance during the conversion of NADPH to NADP^+^ was recorded spectrophotometrically at 340 nm for 3 min. The GSH-Px activity for tissues was expressed as mmol GSH oxidized/min per mg protein.

#### 2.7.10. Determination of Protein Content

The protein content of tissue homogenates was determined by the Lowry protein assay using bovine serum albumin as the standard [27].

### 2.8. Statistical Analysis

The Graph Pad (ISI Software, Philadelphia, PA, USA) computer program was used to conduct regression analysis and to plot collected data. Data are expressed as the mean ± SEM. Results were assessed using one-way ANOVA followed by Tukey-Kramer multiple comparisons tests using Graph Pad Instat (Version 3.06; Graph Pad Software La Jolla, CA, USA). *P* < 0.05 was used as the criterion for significance.

## 3. Results

### 3.1. In Vitro Antioxidant of SMV

The effects of various amounts of SMV on the peroxidation of linoleic acid emulsion are shown in Table 1. The antioxidant activity of SMV in the concentration of 40 *μ*g/mL and 80 *μ*g/mL was greater than that of *α*-tocopherol 80 *μ*g/mL and showed 60.2% and 98.3% inhibition on peroxidation of linoleic acid, respectively, greater than that of *α*-tocopherol (46.2%). The antioxidant activity of SMV in the concentration of 20 *μ*g/mL was close to that of *α*-tocopherol and showed 41.5% inhibition on peroxidation of linoleic acid. Also, Table 1 illustrates a significant (*P* < 0.05) decrease in the concentration of DPPH radical due to the scavenging ability of standards and SMV in a concentration-dependant manner. The scavenging effect of SMV and standards on the DPPH radical decreased in the order of BHA > *α*-tocopherol > SMV. These results indicated that SMV and standards have a noticeable effect on scavenging free radical. Free radical scavenging activity also increased with increasing concentration of SMV in a concentration-dependant manner.

### 3.2. Fluid and Food Intake, Bodyweight, and Organ Weight

Table 2 shows significant differences in fluid and food intakes and bodyweight gain between control and diabetic rats. Increased fluid and food intakes and decreased bodyweight were observed in diabetic rats compared with control rats. Administration of SMV or glibenclamide tended to increase bodyweight to that seen in untreated control rats and the effect was more pronounced in the group of rats treated with glibenclamide. There was no significant change in control rats treated only with SMV.

### 3.3. Blood Glucose, Total Hb, and HbA1_c_

Levels of blood glucose, total Hb, and HbA1_c_ in control and diabetic rats are given in Table 3.

The fasting blood glucose levels and HbA1_c_ were significantly higher in diabetic animals when compared to control rat values, whereas Hb levels were decreased significantly in diabetic rats compared to control rat values. Treatment of diabetic rats with SMV nonsignificantly increased total Hb and significantly (*P* < 0.05) decreased blood glucose level and HbA1_c_ compared to untreated diabetic rat values. On the other hand, glibenclamide significantly reduced fasting blood glucose level and HbA1_c_ when compared with untreated diabetic animals (*P* < 0.001).

### 3.4. Plasma Lipid Profile

In diabetic rats, there was a significant increase (*P* < 0.001) in TC and TG levels by 42 and 124%, respectively, and significant decrease in HDL-C. Oral administration of SMV significantly decreased the levels of TC and TG and increased the levels of HDL-C in diabetic rats compared to untreated diabetic ones. Furthermore, results obtained following treatment with SMV were comparable to those obtained following glibenclamide treatment (Table 4).

### 3.5. Serum Creatinine, BUN, and Urine Protein

**Figure 1:**
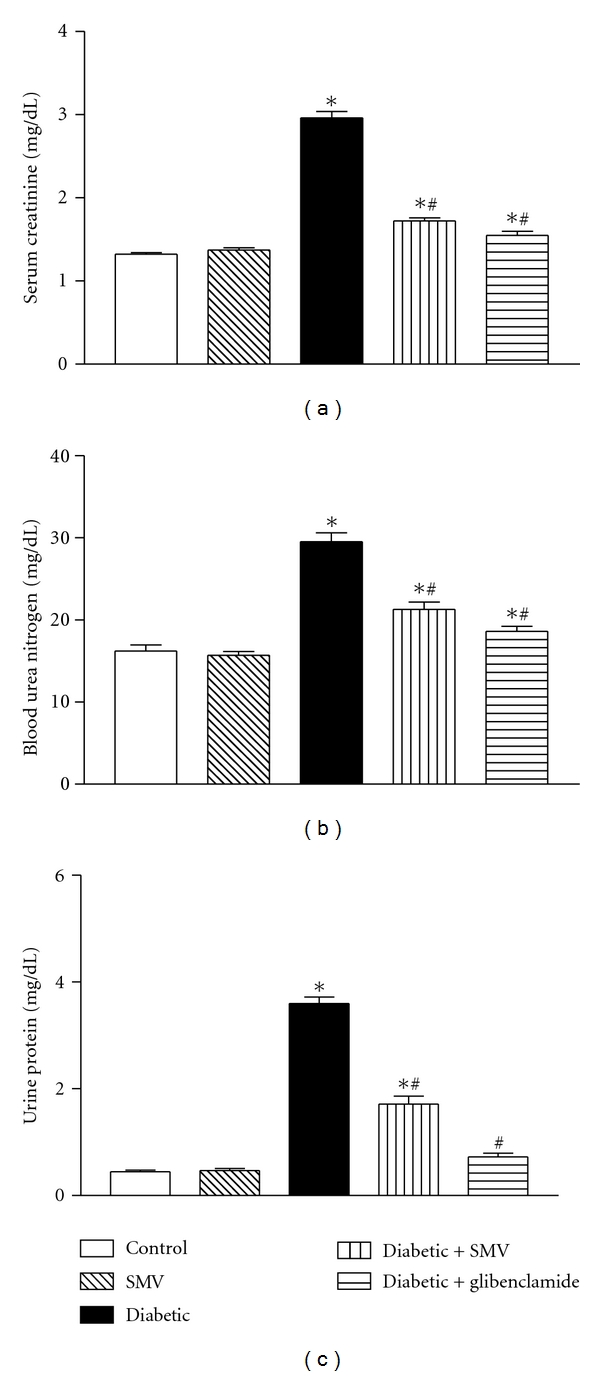
Effect of simvastatin (SMV) and glibenclamide treatment on serum creatinine (a), blood urea (b), and urinary protein (c), in normal and streptozotocin-induced diabetic rats. Data are expressed as means ± SEM (*n* = 8). *Significantly different from control group (*P* < 0.01). ^#^Significantly different from diabetic-untreated group (*P* < 0.01).

Figure 1 shows a significant increase (*P* < 0.05) in the serum creatinine, BUN, and urine protein in untreated diabetic rats when compared with control group. STZ induced almost a twofold increase in the creatinine and urea levels and an eightfold increase in the urine protein levels over the controls rats. All the indices were reduced to near control levels when the SMV was administered to the untreated diabetic rats. In the case of control and SMV only treated rats, the levels of the abovementioned parameters remained unaltered.

### 3.6. Serum ALT, AST, and Total Bilirubin

**Figure 2:**
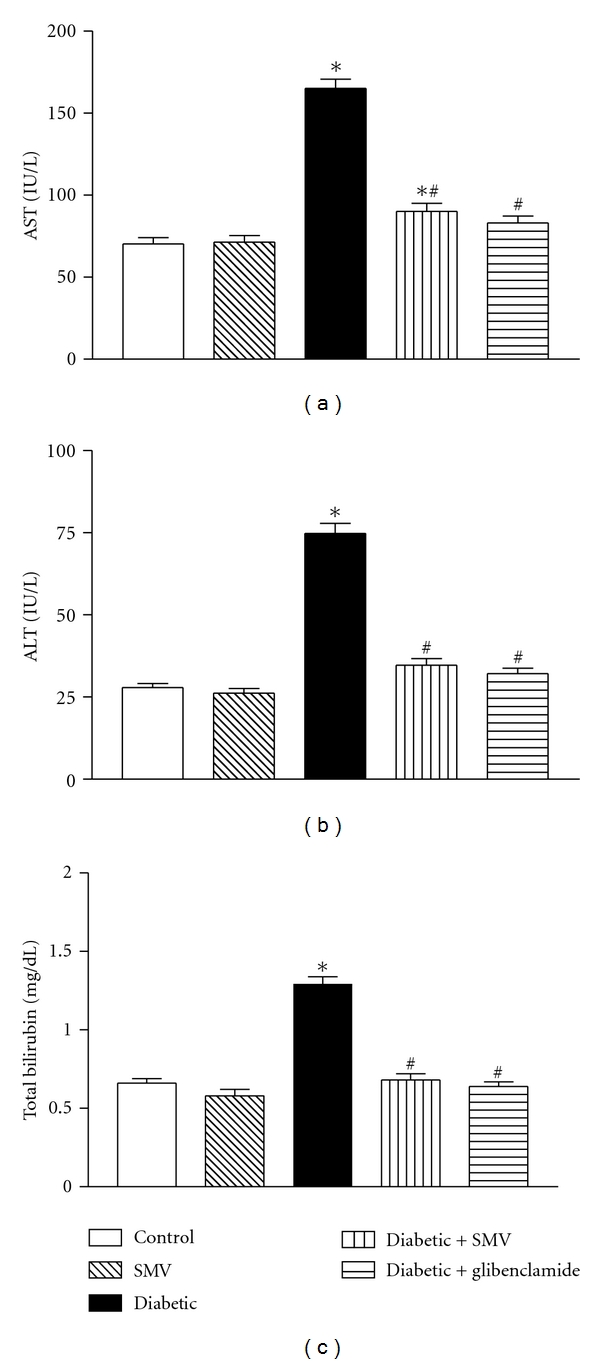
Effect of simvastatin (SMV) and glibenclamide treatment on (a) serum aspartate aminotransferase (AST), (b) alanine aminotransferase (ALT) and (c) total bilirubin in normal and streptozotocin-induced diabetic rats. Data are expressed as means ± SEM (*n* = 8). *Significantly different from control group (*P* < 0.01). ^#^Significantly different from diabetic-untreated group (*P* < 0.01).

The effect of SMV and glibenclamide on STZ-induced liver damage in rats with reference to the changes in the level of AST, ALT, and total bilirubin is shown in Figure 2. Diabetic rats showed significant increase in the levels of AST, ALT and total bilirubin as compared to the normal control group, whereas blood samples analysis from the animals treated with SMV or glibenclamide showed significant decrease in the levels of serum marker enzymes and total bilirubin to the near normal value. 

### 3.7. Plasma Nonenzymatic Antioxidants

**Figure 3:**
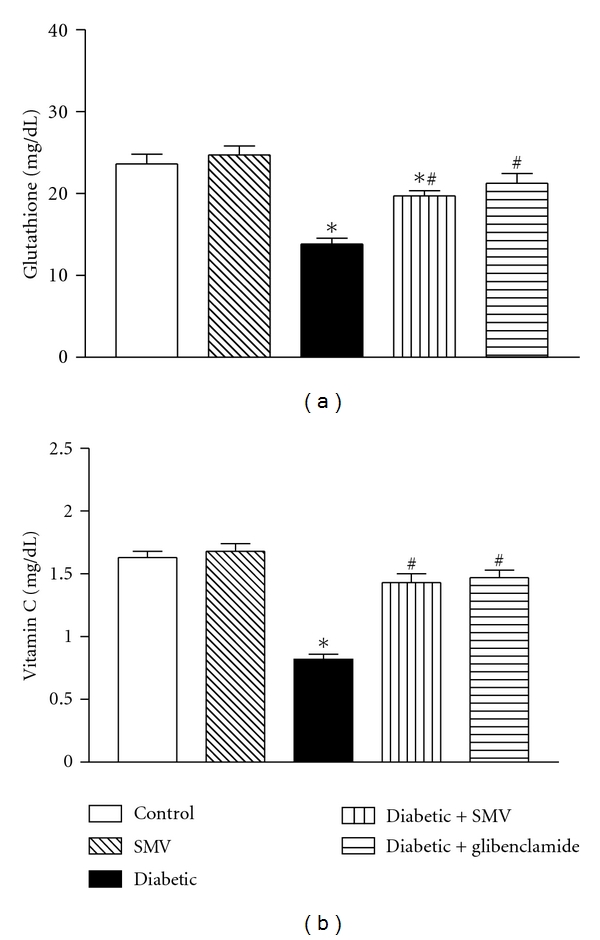
Effect of simvastatin (SMV) and glibenclamide treatment on (a) plasma glutathione and (b) vitamin C in normal and streptozotocin-induced diabetic rats. Data are expressed as means ± SEM (*n* = 8). *Significantly different from control group (*P* < 0.01). ^#^Significantly different from diabetic-untreated group (*P* < 0.01).

The levels of nonenzymatic antioxidants in normal and diabetic rats are given in Figure 3. There was a significant (*P* < 0.05) decrease in the levels of GSH (13.8 ± 0.78 versus 23.6 ± 1.72 mg/dL) and vitamin C (0.82 ± 0.06 versus 1.63 ± 0.11 mg/dL) in diabetic control rats than normal rats. Oral administration of SMV and glibenclamide to diabetic rats leads to a significant (*P* < 0.05) increase in the plasma levels of GSH and vitamin C.

### 3.8. Reduced GSH in Liver and Kidney Homogenates

**Figure 4:**
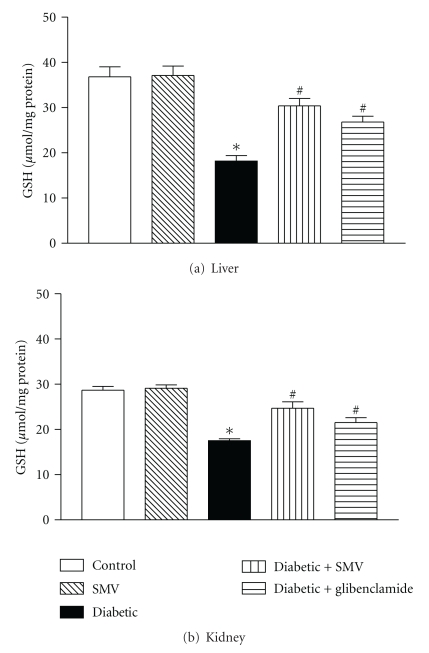
Effect of simvastatin (SMV) and glibenclamide treatment on (a) liver glutathione and (b) kidney glutathione in normal and streptozotocin-induced diabetic rats. Data are expressed as means ± SEM (*n* = 8). *Significantly different from control group (*P* < 0.01). ^#^Significantly different from diabetic-untreated group (*P* < 0.01).

Figures 4(a) and 4(b) show the GSH content in liver and kidney homogenates of control and diabetic rats. There was a significant decrease in the concentration of GSH in the liver and kidney homogenates (50 and 36%, resp.) in diabetic rats compared with control rats. Administration of SMV and glibenclamide increased the GSH content in the liver (74 and 44%, resp.) and kidney homogenates (35 and 18%, resp.) of the diabetic group of rats. The effect was more pronounced in the group of rats treated with SMV and not significant in the groups treated with glibenclamide (*P* > 0.05).

### 3.9. LPO

**Figure 5:**
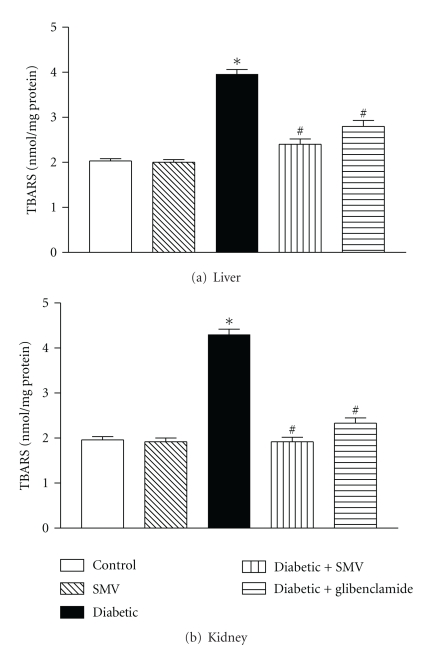
Effect of simvastatin (SMV) and glibenclamide treatment on liver thiobarbituric acid reactive substances (TBARS) in liver (a) and kidney (b) in normal and streptozotocin-induced diabetic rats. Data are expressed as means ± SEM (*n* = 8). *Significantly different from control group (*P* < 0.01).

Liver and kidney TBARS levels, an index of LPO, were higher in diabetic rats (3.96 ± 0.36 and 5.14 ± 0.12 nmol/mg protein, resp.) compared with control rats (2.03 ± 0.14 and 1.96 ± 0.11 nmol/mg protein, resp.) and markedly decreased (*P* < 0.001) by SMV. Level of TBARS in liver and kidney had no significant change in healthy rats treated with SMV; see Figures 5(a) and 5(b).

### 3.10. Antioxidant Enzymes

**Figure 6:**
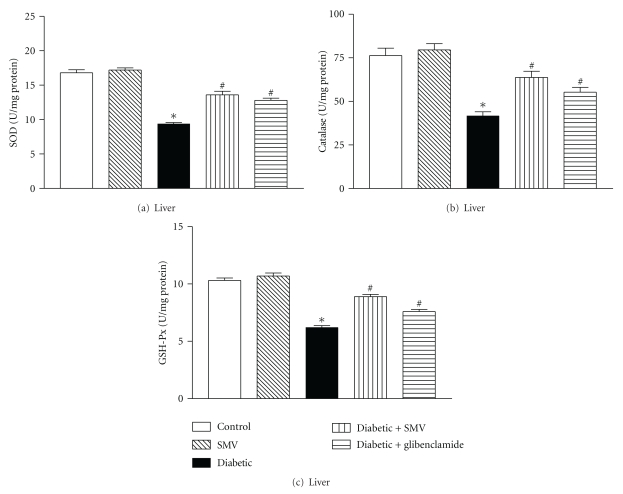
Effect of simvastatin (SMV) and glibenclamide treatment on liver enzymatic antioxidants: (a) SOD, (b) CAT, and (c) GSH-Px in normal and streptozotocin-induced diabetic rats. Data are expressed as means ± SEM (*n* = 8). SOD: superoxide dismutase: CAT, catalase; GSH-Px: glutathione peroxidase. *Significantly different from control group (*P* < 0.01). ^#^Significantly different from diabetic-untreated group (*P* < 0.01).

**Figure 7:**
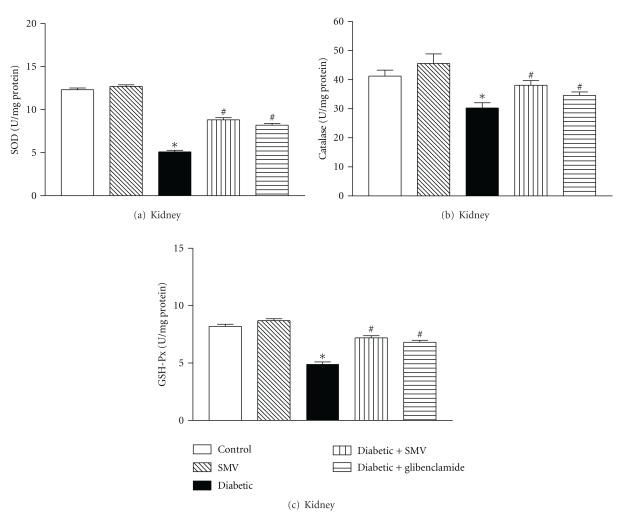
Effect of simvastatin (SMV) and glibenclamide treatment on kidney enzymatic antioxidants: (a) SOD, (b) CAT, and (c) GSH-Px in normal and streptozotocin-induced diabetic rats. Data are expressed as means ± SEM (*n* = 8). SOD: superoxide dismutase; CAT: catalase; GSH-Px: glutathione peroxidase. *Significantly different from control group (*P* < 0.01). ^#^Significantly different from diabetic-untreated group (*P* < 0.01).

Figures 6 and 7 show SOD, CAT, and GSH-Px activities in liver and kidney homogenates of control and diabetic rats. In the diabetic group, there was a significant reduction in SOD, CAT, and GSH-Px activities in the liver (64, 49, and 40%, resp.) and kidney (43, 34, and 39%, resp.) homogenates compared with the control group. Treatment with SMV and glibenclamide increased SOD, CAT, and GSH-Px activity in diabetic rats.

## 4. Discussion

Diabetes mellitus is a highly prevalent chronic illness and it has been reported that increased oxidative stress may play a role in the pathogenesis and progression of diabetic tissue damage [39, 40]. Chronic hyperglycemia in diabetic patients or animals can cause oxidative stress, depleting the activity of the antioxidative defense system and resulting in elevated levels of oxygen-free radicals [6]. Consequences of oxidative stress induce the production of highly ROS that are toxic to cells, particularly the cell membrane in which these radicals interact with the lipid bilayer and produce lipid peroxides and lead to organs oxidative damage [41]. Reduced oxidative stress in the diabetic condition had been observed in experimental animals following the administration of certain antioxidants [42].

 In the current study, administration of STZ resulted in a significant increase in the blood glucose level. Persistent hyperglycemia results in glycation of Hb that leads to the formation of HbA1_c_ [43]. The observed increase in the levels of HbA1_c_ with a concomitant decrease in Hb in the experimental diabetic rats implies the oxidation of sugars, extensive damage to both sugars and proteins in the circulation, and reinforcing the cycle of oxidative stress and damage. Inouye et al. have reported a significant correlation between glycated hemoglobin and different markers of lipid peroxidation [44, 45]. Agents with antioxidant or free radical scavenging power have been shown to inhibit oxidative reactions associated with glycation [46]. In this regard treatment with SMV significantly reversed the imbalance in the oxidative stress status. The antidiabetic effect observed in the present study may be attributed to pleotropic effect of statin as improvement in insulin signaling pathway, protection of *β*-cell from oxidative stress, and increase in insulin release [47, 48]. Recently, it is proven that statins can inhibit dipeptidyl peptidase IV (DPP-IV) [49]. DPP-IV is serine protease, responsible for degradation of Glucagon like peptide 1 (GLP-1) and glucose-dependent insulinotropic polypeptide (GIP). GLP-1 and GIP play important role in glucose homeostasis as both are responsible for 70% of insulin secretion after meal by inhibiting DPP-IV. Therefore statins can increase insulin secretion.

In the present study, lipid profile markers, such as TC, TG, and HDL-C, further confirm that there is a strong correlation between oxidative stress and diabetes occurrence. Increased fasting and postprandial plasma levels of TG, free fatty acids, and cholesterol are common in diabetes and they are known to generate ROS [50, 51]. Antioxidants such as resveratrol, vitamin C, and vitamin E have been reported to reduce STZ-induced oxidative damage [5, 52]. Similarly, we observed that administration of SMV significantly restored abnormal levels of lipid profile markers in blood in diabetic rats. This suggests that SMV may improve lipid dysfunction of diabetic rats and retard development of diabetic complications. Our results are in agreement with previous reports [19, 53].

 Elevated activities of serum AST, ALT, and total bilirubin are a common sign of liver disease and are observed more frequently among people with diabetes than in the general population [54]. SMV treatment prevented the increase in these enzymatic activities in serum that was caused by STZ administration. Our results are in agreement with those of Imaeda et al. [55] who also found that antioxidants inhibited the increase in serum levels of AST and ALT in STZ-treated mice. Increased serum levels of urea and creatinine, indicators of impaired renal function [19], observed in the diabetic rats might indicate renal damage. Treatment with SMV significantly decreased serum creatinine and urea. These data suggest that SMV may help in the repair of renal damage.

 During diabetes, an increased oxidative stress in certain tissues may lead to a rise in the rate of LPO [8]. The formation of the lipid peroxide product, TBARS, was measured in tissue as an index of increased LPO in diabetic liver and kidneys [56]. In the present study, the increased TBARS content of diabetic rats suggests that peroxidative injury may be involved in the development of diabetic complications. TBARS levels in liver and kidneys were significantly decreased in the SMV and glibenclamide-treated groups compared to the diabetic control rats. Recently, agents with antioxidant or free radical scavenging power have been shown to inhibit oxidative reactions associated with LPO [5, 52]. The above result suggests that the SMV may exert antioxidant effects and protect the tissues from LPO.

 Nonenzymatic antioxidants such as GSH and vitamin C play an excellent role in preventing the cells from oxidative damage. GSH is an intracellular thiol rich tripeptide, which plays a major role in the protection of cells and tissue structures [57]. GSH is required for the recycling of vitamin C [58] and acts as a substrate for GSH-Px that is involved in preventing the deleterious effect of free radicals [59]. In our study, diabetic rats exhibited decreased level of GSH, which might be due to increased utilization of GSH for scavenging free radicals by GSH-Px. Administration of SMV reversed GSH level in plasma of diabetic rats, which could be due to the low peroxidisability and thus its low utilization. The present findings of decreased GSH in liver and kidneys are consistent with the studies of Panda et al. [56].

 Vitamin C is a well-known physiological hydrophilic antioxidant in plasma, because it disappears faster than other antioxidants when plasma is exposed to ROS [60]. The observed significant decrease in the level of plasma vitamin C could be caused by increased utilization of vitamin C as an antioxidant defense against ROS or by a decrease in GSH, which is required for the recycling of vitamin C. Treatment with SMV brought vitamin C to near normal levels which could be as a result of decreased membrane damage as evidenced by the antioxidant nature.

Simvastatin is a lipophilic compound that may have more potent effects on extrahepatic sites [61]. It has a short half-life time of about 2 h and is cleared by extensive metabolism in the intestinal gut and liver by cytochrome (CYP) 3A [62]. In the present study, SMV showed antioxidant effect both in vitro and in vivo. ROS-induced oxidative damage has been implicated in the pathogenesis of several disorders, including diabetes mellitus [63]. Oxidative stress is the imbalance between production and removal of ROS. Increased oxidative stress, which contributes substantially to the pathogenesis of diabetic complications, is the consequence of either enhanced ROS production or attenuated ROS-scavenging capacity. Several studies have demonstrated both lower nonenzymatic antioxidant levels and enzymatic antioxidant activities in streptozotocin-induced diabetic rats [9, 56]. Recently, Sefi et al. [64] have reported an elevated LPO and lowered antioxidants in streptozotocin-induced diabetes mellitus.

Enzymatic antioxidants (SOD, CAT, and GSH-Px) form the first line of the antioxidant defense mechanism to protect the organism from ROS-mediated oxidative damage [58]. In the current study, SOD, CAT and GSH-Px showed lower activities in liver and kidney during diabetes and the results agree well with the earlier published data [65, 66]. The decreased activities of SOD, CAT, and GSH-Px may be a response to increased production of H_2_O_2_ and O_2_^.−^ by the auto-oxidation of the excess of glucose and nonenzymatic glycation of proteins [67]. Pigeolet et al. [68] have reported the partial inactivation of these enzyme activities by hydroxyl radicals and hydrogen peroxide. The decreased activity of SOD and CAT could also be due to their decreased protein expression levels in the diabetic condition, as recently reported in liver [69]. The decreased GSH-Px activity represents a compensatory mechanism to degrade H_2_O_2_. Treatment of the diabetic rats with SMV restored the altered antioxidant enzyme activities significantly (*P* < 0.001).

 In conclusion, the present investigation showed that SMV may possess an antioxidant activity and it also protects LPO and enhances its effect on enzymatic antioxidant (SOD, CAT, and GSH-Px) and nonenzymatic antioxidant (GSH and vitamin C) defense. This activity contributes to the protection against oxidative damage in STZ-induced diabetes.

## Figures and Tables

**Table 1:** In vitro total antioxidant and free radical scavenging activities of simvastatin (SMV), buthylated hydroxyanisole (BHA), and *α*-tocopherol (*α*-Toc).

Groups	Total antioxidant activity (Inhibition of lipid peroxidation %)^a^	Free radical scavenging activity (DPPH^•^ *μ*M)^b^
*α*-Toc (80 *μ*g/mL)	46.2	76
BHA (80 *μ*g/mL)	93.7	83
SMV (20 *μ*g/mL)	41.2	38
SMV (40 *μ*g/mL)	60.2	44
SMV (80 *μ*g/mL)	98.3	72

Data are the mean ± SEM for eight animals in each group.

^a^The antioxidant activity of different doses of simvastatin (20–80 *μ*g/mL) was determined by the thiocyanate method. The peroxide values were determined by reading the absorbance at 500 nm after reaction with FeCl_2_ and thiocyanate. ^b^Free radical scavenging activity of different doses of SMV, BHA, and *α*-tocopherol by 1,1-diphenyl-2-picrylhydrazyl radicals.

**Table 2:** Effect of simvastatin (SMV) and glibenclamide supplementation on fluid and food intake and body weight of rats in the different experimental groups.

Groups	Fluid intake (mL/day)	Food intake (g/day)	Weight gain (g/day)
Control	28 ± 3	15.3 ± 0.2	4.90 ± 0.03
SMV	33 ± 2	18.2 ± 0.42	4.41 ± 0.06
Diabetic	96 ± 5*	25.7 ± 0.3*	2.96 ± 0.04*
Diabetic + SMV	46 ± 4^∗#^	22.1 ± 0.5*	3.30 ± 0.05*
Diabetic + Glibenclamide	36 ± 2^∗#^	23.0 ± 0.2*	3.71 ± 0.03^∗#^

Data are the mean ± SEM for eight animals in each group.

*P < 0.001 compared with the control group.

^#^ *P* < 0.05 compared with the untreated diabetic group.

**Table 3:** Effect of simvastatin (SMV) and glibenclamide supplementation on blood glucose, haemoglobin (Hb) and glycosylated haemoglobin (HbA1_c_) of rats in different experimental groups.

Groups	Blood glucose (mg/dL)	Hb (g/dL)	HbA1_c_ (% Hb)
Control	84.3 ± 4.2	12.8 ± 0.41	5.8 ± 0.32
SMV	87.5 ± 3.6	13.0 ± 0.36	6.1 ± 0.21
Diabetic	336 ± 9.8*	9.8 ± 0.43*	12.7 ± 0.5*
Diabetic + SMV	260 ± 7.3^∗#^	10.6 ± 0.52	10.2 ± 0.33^∗#^
Diabetic + Glibenclamide	127.5 ± 6.4^∗#^	12.3 ± 0.48^#^	6.7 ± 0.03^#^

Data are the mean ± SEM for eight animals in each group.

*P < 0.001 compared with the control group.

^#^ *P* < 0.05 compared with the untreated diabetic group.

**Table 4:** Effect of simvastatin (SMV) and glibenclamide supplementation on serum total cholesterol, high-density lipoprotein-cholesterol (HDL-C) and triglycerides for rats in different experimental groups.

Groups	Total cholesterol (mg/dL)	HDL-C (mg/dL)	Triglycerides (mg/dL)
Control	77.4 ± 4.4	42.5 ± 1.3	57.3 ± 1.8
SMV	70.8 ± 3.1	44.6 ± 1.6	50.1 ± 2.1
Diabetic	110.3 ± 5.1*	31.2 ± 2.2*	128.6 ± 3.6*
Diabetic + SMV	80.2 ± 2.9^∗#^	55.7 ± 2.8^∗#^	61.2 ± 2.4^#^
Diabetic + Glibenclamide	89.3 ± 3.6^∗#^	45.3 ± 1.9^#^	68.7 ± 3.6^∗#^

Data are the mean ± SEM for eight animals in each group.

*P < 0.001 compared with the control group.

^#^ *P* < 0.05 compared with the untreated diabetic group.
