# Corrigendum to “Protective Effects of Simvastatin, a Lipid Lowering Agent, Against Oxidative Damage in Experimental Diabetic Rats”

**DOI:** 10.1155/2020/4536827

**Published:** 2020-12-16

**Authors:** Ahmed M. Mohamadin, Ahmed A. Elberry, Hala S. Abdel Gawad, Gehan M. Morsy, Fahad A. Al-Abbasi

**Affiliations:** ^1^Department of Chemistry for Health Sciences, Deanery of Academic Services, Health Sciences Track, Taibah University, Al-Madinah, Saudi Arabia; ^2^Biochemistry Department, Faculty of Pharmacy, Al-Azhar University, Nasr City, Cairo, Egypt; ^3^Department of Clinical Pharmacy, Faculty of Pharmacy, King Abdulaziz University, Jeddah, Saudi Arabia; ^4^Department of Physiology, College of Medicine, Taibah University, Al-Madinah, Saudi Arabia; ^5^Department of Biochemistry and Nutrition, Women Faculty for Arts, Science and Education, Ain Shams University, Cairo, Egypt; ^6^Department of Biochemistry, Faculty of Science, King Abdulaziz University, Jeddah, Saudi Arabia

In the article titled “Protective Effects of Simvastatin, a Lipid Lowering Agent, Against Oxidative Damage in Experimental Diabetic Rats” [1], author “Gehan M. Morsy” was affiliated to “Biochemistry Department, Applied Science College, Taibah University, Al-Madinah, Saudi Arabia,” which is incorrect. The correct affiliation for this author is:

“Department of Biochemistry and Nutrition, Women Faculty for Arts, Science and Education, Ain Shams University, Cairo, Egypt”
